# Waist circumference, abdominal obesity, and depression among overweight and obese U.S. adults: national health and nutrition examination survey 2005-2006

**DOI:** 10.1186/1471-244X-11-130

**Published:** 2011-08-11

**Authors:** Guixiang Zhao, Earl S Ford, Chaoyang Li, James Tsai, Satvinder Dhingra, Lina S Balluz

**Affiliations:** 1 Division of Adult and Community Health, National Center for Chronic Disease Prevention and Health Promotion, Atlanta, GA 30341, USA; 2 Division of Behavioral Surveillance, Public Health Surveillance Program Office, Office of Surveillance, Epidemiology and Laboratory Services, Centers for Disease Control and Prevention, Atlanta, GA 30341, USA

**Keywords:** abdominal obesity, depressive symptoms, PHQ-9 diagnostic algorithm, waist circumference

## Abstract

**Background:**

Obesity is associated with an increased risk of mental illness; however, evidence linking body mass index (BMI)-a measure of overall obesity, to mental illness is inconsistent. The objective of this study was to examine the association of depressive symptoms with waist circumference or abdominal obesity among overweight and obese U.S. adults.

**Methods:**

A cross-sectional, nationally representative sample from the 2005-2006 National Health and Nutrition Examination Survey was used. We analyzed the data from 2,439 U.S. adults (1,325 men and 1,114 nonpregnant women) aged ≥ 20 years who were either overweight or obese with BMI of ≥ 25.0 kg/m^2^. Abdominal obesity was defined as waist circumference of > 102 cm for men and > 88 cm for women. Depressive symptoms (defined as having major depressive symptoms or moderate-to-severe depressive symptoms) were assessed by the Patient Health Questionnaire-9 diagnostic algorithm. The prevalence and the odds ratios (ORs) with 95% confidence intervals (CIs) for having major depressive symptoms and moderate-to-severe depressive symptoms were estimated using logistic regression analysis.

**Results:**

After multivariate adjustment for demographics and lifestyle factors, waist circumference was significantly associated with both major depressive symptoms (OR: 1.03, 95% CI: 1.01-1.05) and moderate-to-severe depressive symptoms (OR: 1.02, 95% CI: 1.01-1.04), and adults with abdominal obesity were significantly more likely to have major depressive symptoms (OR: 2.18, 95% CI: 1.35-3.59) or have moderate-to-severe depressive symptoms (OR: 2.56, 95% CI: 1.34-4.90) than those without. These relationships persisted after further adjusting for coexistence of multiple chronic conditions and persisted in participants who were overweight (BMI: 25.0-< 30.0 kg/m^2^) when stratified analyses were conducted by BMI status.

**Conclusion:**

Among overweight and obese U.S. adults, waist circumference or abdominal obesity was significantly associated with increased likelihoods of having major depressive symptoms or moderate-to-severe depressive symptoms. Thus, mental health status should be monitored and evaluated in adults with abdominal obesity, particularly in those who are overweight.

## Background

Obesity continues to be a cause of public concern in the United States and worldwide [1]. The prevalence of obesity, defined as a body mass index (BMI) of ≥ 30 kg/m^2^, was 32% in the United States during 2001-2004 and increased slightly to 34% during 2005-2006 [2]. The health impact of obesity is tremendous, as shown by an increased risk for multiple chronic diseases and conditions including hypertension, diabetes, hypercholesterolemia, coronary heart disease, asthma, arthritis, cancers, and many others [3-5]. In addition, obesity, especially abdominal obesity, is associated with increased all-cause, cardiovascular, or cancer mortality [6].

In addition to the broad range of obesity-related physiologic outcomes, obesity is associated with an increased risk for a number of mental disorders (i.e., depression, bipolar disorder, panic disorder, anxiety, or many others) [7-15] that have a substantial impact on public health (e.g., associated with great burden of diseases and increased mortality, disability, and reduced quality of life) [16,17]. However, other studies showed that BMI was not [18-22] or was even inversely associated with some forms of mental illness [20,23]. A possible explanation for these inconsistent results is that BMI as a measure of overall obesity does not account for varying proportions of muscle mass, bone, and fat, or the distribution of body fat. In fact, studies have consistently shown that abdominal visceral fat is more pathogenic than subcutaneous fat on metabolic risk profiles [24,25] and that fat distribution (central adiposity vs general obesity, or visceral vs subcutaneous fat) is differentially associated with depressive symptoms [20,26,27]. Waist circumference, frequently used as a simple, inexpensive measure of central obesity in population-based studies, has been shown to be associated with depression in some studies [28,29] but not in others [21,23]. By using a large nationally representative sample, we sought to examine whether abdominal obesity measured by waist circumference was associated with depressive symptoms among overweight and obese adults after taking into consideration multiple risk factors including demographic characteristics, lifestyle factors, and coexistence of multiple chronic conditions. In this study, we only focused on adults who were overweight and obese because abdominal obesity is more physiologically or psychologically relevant in this population than in people who are underweight or normal weight, among whom depressive symptoms are unrelated to visceral fat [27]. Our study makes a unique contribution to the literature by using a larger, population-based, and nationally representative sample including both men and women with objective measures of overall and central obesity, which is rare for epidemiologic research.

## Methods

### Participants and measures

A cross-sectional, nationally representative sample from the National Health and Nutrition Examination Survey (NHANES) 2005-2006 was obtained using a multistage stratified sampling design. Survey participants were initially interviewed at home and were then invited to a mobile examination center, where they received various examinations and provided blood samples for laboratory tests. All procedures involving human subjects were approved by the Research Ethics Review Board of the National Center for Health Statistics, Centers for Disease Control and Prevention. Written informed consent was obtained from all participants. Details about the NHANES survey design and operation are available elsewhere [30].

We examined interview and laboratory data from participants aged ≥ 20 years who were noninstitutionalized U.S. civilian. Data on anthropometric measurements were collected by trained health technicians [31]. BMI was calculated from measured weight and height following a standardized protocol. Participants with a BMI of ≥ 25.0 kg/m^2 ^(either overweight or obese) were included in this study. Waist circumference was measured at a point immediately above the iliac crest on the midaxillary line at minimal respiration to the nearest 0.1 cm [31,32]. Abdominal obesity was defined as waist circumference of > 102 cm for men and > 88 cm for women [6].

Participants' depressive symptoms were assessed by using the Patient Health Questionnaire-9 (PHQ-9) diagnostic algorithm, which has been described in detail elsewhere [33]. Specifically, participants were asked about how often over the last 2 weeks they had experienced each of the following symptoms: 1) little interest or pleasure in doing things; 2) feeling down, depressed, or hopeless; 3) trouble falling or staying asleep or sleeping too much; 4) feeling tired or having little energy; 5) having a poor appetite or overeating; 6) feeling bad as a failure or having let themselves or their family down; 7) having trouble concentrating on things such as reading the newspaper or watching TV; 8) moving or speaking so slowly that other people could have noticed, or being so fidgety or restless that they had been moving around a lot more than usual; and 9) having thoughts of suicidality or self-injury in some way. Participants were defined as having major depressive symptoms if they had at least five of the nine PHQ-9 criteria for ≥ 7 days (or ≥ several days for "having thoughts of suicidality or self-injury") in the past 2 weeks, one of which must be "loss of interest or pleasure in doing things" or "feel down, depressed, or hopeless" for ≥ 7 days in the past 2 weeks [34]. Alternatively, participants' responses to each item were scored as 0 point for "not at all", 1 point for "having the symptoms for several days", 2 points for "having the symptoms for more than half the days", and 3 points for "having the symptoms for nearly every day". Their scores for each item were then added to produce a total depression severity score, and the cutoff point of ≥ 10 was used to identify participants as having moderate-to-severe depressive symptoms [34,35]. The PHQ-9 has been shown to provide valid measurements of depression in the general population as well as in patients with diabetes, coronary artery disease, and heart failure. Using a structured mental health professional interview as the criterion standard, a PHQ-9 score of ≥ 10 had a sensitivity of 88% and a specificity of 88% for major depression, and, regardless of diagnostic status, typically represents clinically significant depression [34-36].

Socio-demographic variables used in the analyses included age, sex, race/ethnicity (non-Hispanic white, non-Hispanic black, and other including Mexican American, non-Mexican American, and any other races), educational status (< high school diploma, high school graduate, and > high school diploma), and family poverty-income ratio (calculated as a ratio of family income to poverty threshold). Smoking status was reflected by serum concentrations of cotinine which were measured by an isotope dilution-high performance liquid chromatography/atmospheric pressure chemical ionization tandem mass spectrometry (Perkin-Elmer Sciex Co, Norwalk, CT). Physical activity was calculated as an average daily metabolic equivalent (MET)-hour index that summed transportation, household, and leisure-time physical activity. Alcohol consumption was calculated as the average number of daily drinks for each participant. Heavy alcohol drinking was defined as having > 2 drinks per day in men and having > 1 drink per day in women. The number of chronic conditions including hypertension, diabetes, coronary heart disease, stroke, arthritis, asthma, chronic bronchitis, chronic renal disease, and cancer was also included as a covariate. Most of these conditions were assessed by asking participants whether they had ever been told by a healthcare professional that they had diabetes, coronary heart disease, stroke, arthritis, or cancer, or whether they still had asthma and chronic bronchitis. For blood pressure, up to four readings of systolic and diastolic blood pressure were obtained from participants in the mobile examination centers. The average of the last two measurements of systolic or diastolic blood pressure for participants who had three or four measurements, the last measurement for participants with only two measurements, and the only measurement for participants who had one measurement were used to establish high blood pressure status. According to the Joint National Committee on Prevention, Detection, Evaluation, and Treatment of High Blood Pressure reports [37], participants who were on antihypertension medications or had systolic blood pressure ≥ 140 mmHg or diastolic blood pressure ≥ 90 mmHg were defined as having hypertension. For kidney disease, we estimated glomerular filtration rate using the CKD-EPI (Chronic Kidney Disease Epidemiology Collaboration) equation [38], and participants with a glomerular filtration rate of < 60 mL/min/1.73 m^2 ^were defined as having chronic renal disease.

### Statistical analysis

From a total of 3,250 adult participants who were overweight or obese, 231 women were excluded because of pregnancy. After further excluding those who had missing values for any of the study variables, 2,439 participants (1,325 men and 1,114 nonpregnant women) remained in our analyses. The prevalence of having major depressive symptoms or moderate-to-severe depressive symptoms (PHQ-9 score ≥ 10) was age-standardized to the 2000 projected U.S. population. The odds ratios (ORs) with 95% confidence intervals (CIs) for major depressive symptoms or moderate-to-severe depressive symptoms were estimated by conducting logistic regression analyses to test associations between depressive symptoms and waist circumference (used as a continuous variable) or abdominal obesity (used as a categorical variable) while controlling for covariates which included demographic characteristics (age, sex, race/ethnicity, education, and family poverty-income ratio), lifestyle factors (serum concentrations of cotinine, physical activity, and heavy alcohol drinking), and coexistence of multiple chronic conditions (hypertension, diabetes, coronary heart disease, stroke, arthritis, asthma, chronic bronchitis, chronic kidney disease and cancer). SUDAAN (Software for the Statistical Analysis of Correlated Data, Release 9.0, Research Triangle Institute, Research Triangle Park, NC) was used to account for the complex sampling design.

## Results

Overall, the unadjusted and age-adjusted prevalence of having major depressive symptoms among adults who were overweight or obese was 2.5% (95% CI: 1.7-3.7%) and 2.3% (95% CI: 1.6-3.4%), respectively, and was 5.6% (95% CI: 4.4-7.0%) and 5.4% (95% CI: 4.3-6.7%), respectively, for having moderate-to-severe depressive symptoms (PHQ-9 score ≥ 10). Participants' socio-demographic characteristics differed significantly by depressive symptom status except for race/ethnicity (Table 1). Notably, the percentages of adults who were middle-aged (40-< 60 years), female, and obese were significantly higher, whereas the percentages of adults who attained an educational level of > high school diploma or had a poverty-income ratio of ≥ 3 were significantly lower, among participants with major depressive symptoms or moderate-to-severe depressive symptoms than among those without (P < 0.05 for all comparisons). Overall, the percentages of adults with ≥ 3 chronic conditions were higher among participants with major depressive symptoms or moderate-to-severe depressive symptoms than among those without (Table 1). The mean waist circumference among participants with major depressive symptoms or moderate-to-severe depressive symptoms was significantly higher compared to those without depression (P < 0.05).

**Table 1:** Characteristics of overweight and obese study participants by major depressive symptoms or by moderate-to-severe depressive symptoms (PHQ-9 score of ≥ 10), NHANES 2005-2006 *

	n	Major depressive symptoms			Moderate-to-severe depressive symptoms (PHQ-9 score ≥ 10)		
		
		Yes	No	***P-value***†	Yes	No	***P-value***†
N	2,439	68	2,371		152	2,287	
Age (year)				*0.009*			*0.019*
20-< 40	746	19.7 (3.6)	32.5 (1.2)		24.3 (2.8)	32.6 (1.3)	
40-< 60	886	69.3 (4.8)	43.4 (1.5)		57.0 (4.0)	43.3 (1.5)	
≥ 60	807	11.0 (4.4)	24.1 (2.1)		18.7 (3.9)	24.1 (2.1)	
Sex				*< 0.001*			*0.007*
Men	1,325	30.9 (6.2)	54.3 (0.8)		40.8 (4.9)	54.5 (0.9)	
Women	1,114	69.1 (6.2)	45.7 (0.8)		59.2 (4.9)	45.5 (0.9)	
Race‡				*0.249*			*0.152*
NH white	1,215	69.0 (8.3)	73.2 (2.9)		68.1 (5.6)	73.4 (3.0)	
NH black	589	17.5 (4.6)	11.5 (2.0)		16.7 (3.5)	11.4 (2.0)	
Other	635	13.5 (5.2)	15.3 (1.9)		15.2 (4.1)	15.2 (2.0)	
Education				*0.144*			*0.020*
< high school diploma	628	21.6 (5.3)	16.1 (1.6)		18.9 (3.3)	16.1 (1.6)	
high school graduate	608	39.2 (7.6)	25.6 (1.1)		37.7 (4.2)	25.3 (1.1)	
> high school diploma	1,203	39.2 (7.2)	58.2 (1.8)		43.4 (4.3)	58.6 (1.8)	
PIR§				*< 0.001*			*< 0.001*
< 1	393	33.5 (5.9)	9.5 (0.7)		26.9 (2.5)	9.1 (0.7)	
1-< 3	987	45.9 (7.8)	34.2 (2.1)		45.7 (4.9)	33.8 (2.1)	
3-≤5	1,059	20.6 (6.9)	56.3 (2.3)		27.4 (4.0)	57.1 (2.3)	
BMI (kg/m^2^)				*0.037*			*0.008*
25-< 30	1,202	33.0 (5.9)	49.1 (1.6)		34.0 (4.8)	49.6 (1.6)	
≥ 30	1,237	67.0 (5.9)	50.9 (1.6)		66.0 (4.8)	50.4 (1.6)	
No. of chronic conditions**				*0.014*			*< 0.001*
0	966	20.6 (7.0)	42.3 (1.6)		23.2 (4.6)	42.8 (1.5)	
1	621	27.8 (9.0)	26.8 (1.8)		23.9 (5.3)	27.0 (1.8)	
2	401	26.0 (4.6)	16.0 (1.0)		20.1 (3.3)	16.0 (1.0)	
≥ 3	451	25.7 (6.6)	14.9 (1.4)		32.8 (3.2)	14.2 (1.4)	
Waist circumference (cm)	2,439	110.5 (2.2)	105.1 (0.5)	*0.022*	109.1 (1.7)	105.0 (0.5)	*0.019*

*Data expressed as percentages with standard errors in parentheses for all categorical variables and expressed as means with standard errors for waist circumference. †Chi-Square test for categorical variables and Student t-test for continuous variables. ‡NH: non-Hispanic, §PIR: poverty-income ratio. ** Chronic conditions included hypertension, diabetes, coronary heart disease, stroke, arthritis, asthma, chronic bronchitis, chronic renal disease, and cancer.

**Figure 1:**
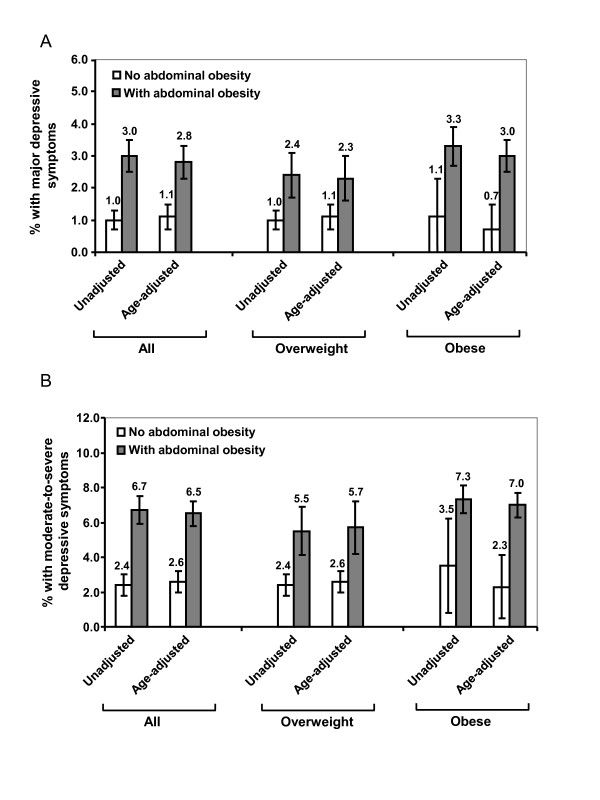
**Unadjusted and age-adjusted prevalence (with standard error) of having major depressive symptoms (A) or having moderate-to-severe depressive symptoms (B) by overall and abdominal obesity among U.S. adults, National Health and Nutrition Examination Survey 2005-2006 (N = 2,439)**.

The unadjusted and age-adjusted prevalence of having major depressive symptoms or moderate-to-severe depressive symptoms was significantly higher among participants with abdominal obesity than among those without abdominal obesity (Figure 1A and 1B, P < 0.001). Stratified analyses on overweight and obese adults yielded similar results (Figure 1). In unadjusted models (Model 1), waist circumference was significantly associated with the presence of both major depressive symptoms and moderate-to-severe depressive symptoms (P < 0.01, Table 2); the relationships persisted after adjusting for socio-demographic variables and lifestyle factors (Model 2). After further adjusting for the coexistence of multiple chronic diseases (Model 3), waist circumference remained significantly associated with the presence of major depressive symptoms (P = 0.031) but was only marginally associated with the presence of moderate-to-severe depressive symptoms (P = 0.074). When abdominal obesity was entered in the models, significant associations between abdominal obesity and depressive symptoms (by all definitions) existed after adjusting for all potential confounders (P ≤ 0.01, Table 2). No significant interactions between sex and waist circumference or between sex and abdominal obesity were observed in fully adjusted models.

**Table 2:** Associations of major depressive symptoms or moderate-to-severe depressive symptoms with waist circumference or abdominal obesity among overweight and obese adults, NHANES 2005-2006 (N = 2,439)

	Odds Ratio*		
	
	Model 1	Model 2	Model 3
**Major depressive symptoms**			
Waist circumference†	1.03 (1.01-1.05)	1.03 (1.01-1.05)	1.03 (1.00-1.05)
*Wald P-value*	*0.002*	*0.005*	*0.031*
			
Abdominal obesity‡			
yes	3.01 (2.00-4.55)	2.18 (1.35-3.59)	1.99 (1.18-3.38)
no	1.00	1.00	1.00
*Wald P-value*	*< 0.001*	*< 0.001*	*0.005*
		
**Moderate-to severe depressive symptoms** **(PHQ score ≥ 10)**			
Waist circumference†	1.02 (1.01-1.04)	1.02 (1.01-1.04)	1.02 (1.00-1.03)
*Wald P-value*	0.003	0.004	0.074
			
Abdominal obesity‡			
yes	2.89 (1.70-4.91)	2.56 (1.34-4.90)	2.26 (1.15-4.44)
no	1.00	1.00	1.00
*Wald P-value*	*< 0.001*	*0.002*	*0.010*

*Model 1: unadjusted, Model 2: adjusted for age, sex, race, education, family poverty-income ratio, serum cotinine concentrations, physical activity and heavy alcohol drinking, Model 3: adjusted for the same set of variables as in Model 2 plus coexistence of the number of chronic conditions (including hypertension, diabetes, coronary heart disease, stroke, arthritis, asthma, chronic bronchitis, chronic kidney disease and cancer).

†Used as continuous variable in the models

‡Used as categorical variable (defined as waist circumference > 108 cm for men and > 88 cm for women)

When data analyses were further stratified by BMI and abdominal obesity status, abdominal obesity remained significantly associated with having major depressive symptoms (OR: 1.67, 95% CI: 1.12-2.50) and marginally associated with having moderate-to-severe depressive symptoms (OR: 2.03, 95% CI: 0.98-4.20) among participants who were overweight. However, the odds ratios between obese people with abdominal obesity and those without were not significant (Table 3).

**Table 3:** Odds ratios (with 95% CIs) of having major depressive symptoms or moderate-to-severe depressive symptoms by overweight/obese and by abdominal obesity among overweight and obese adults, NHANES 2005-2006 (N = 2,439)

		N		Odds Ratio*	
			
			Model 1	Model 2	Model 3
**Major depressive symptoms**					
Overweight	- abdominal obesity	615	1.00	1.00	1.00
	+ abdominal obesity	587	2.38 (1.37-4.12)	1.77 (1.14-2.75)	1.67 (1.12-2.50)
Obese	- abdominal obesity	52	1.10 (0.11-11.12)	0.87 (0.09-8.09)	0.83 (0.09-7.70)
	+ abdominal obesity	1,185	3.36 (1.99-5.66)	2.29 (1.24-4.23)	2.07 (1.03-4.14)
*Wald P-value*			*< 0.001*	*< 0.001*	*< 0.001*
**Moderate-to severe depressive symptoms** **(PHQ score ≥ 10)**					
Overweight	- abdominal obesity	615	1.00	1.00	1.00
	+ abdominal obesity	587	2.41 (1.25-4.67)	2.18 (1.06-4.48)	2.03 (0.98-4.24)
Obese	- abdominal obesity	52	1.49 (0.28-7.80)	1.31 (0.26-6.52)	1.19 (0.23-6.00)
	+ abdominal obesity	1,185	3.28 (1.91-5.65)	2.79 (1.42-5.48)	2.40 (1.18-4.86)
*Wald P-value*			*< 0.001*	*0.014*	*0.062*

*Model 1: unadjusted, Model 2: adjusted for age, sex, race, education, family poverty-income ratio, serum cotinine concentrations, physical activity and heavy alcohol drinking, Model 3: adjusted for the same set of variables as in Model 2 plus coexistence of the number of chronic conditions (including hypertension, diabetes, coronary heart disease, stroke, arthritis, asthma, chronic bronchitis, chronic kidney disease and cancer).

## Discussion

Using a large, population-based sample from NHANES, we found that, among overweight and obese adults, waist circumference and/or abdominal obesity was significantly associated with increased prevalence and likelihood of having major depressive symptoms or moderate-to-severe depressive symptoms, suggesting abdominal obesity is a strong correlate of depression, particularly for adults who were overweight by their BMI status.

The relationship between depression and waist circumference or abdominal obesity as a component of metabolic syndrome has been explored previously in studies examining the associations of metabolic syndrome with mental illness [39-43]. The inconsistent results of these studies may have resulted from differences in the populations being studied, in the measures of depression used, or in the number and type of covariates controlled for across studies. For example, three studies that were conducted in participants aged 35-55 years in London using the 4-item depression subscale of the General Health Questionnaire [39], in participants aged 25-84 years in Australia using the Hospital Anxiety and Depression Scale [40], and in Japanese men aged 20-67 years using the Profile of Mood States of the Likert-scale questionnaire [43] showed a significant association between waist circumference and depression. However, two studies conducted in Finland, one in participants aged 31 years using the Hopkins Symptom Checklist-25 questionnaire [41] and the other in participants aged 36-55 years using the Beck Depression Inventory [42], failed to observe a significant association as did studies conducted in Chinese elderly (aged ≥ 55 years) using the Geriatric Depression Scale-15 items [23] and in participants aged ≥ 25 years in New Zealand using self-reported, physician-diagnosed depression [21]. Moreover, two studies conducted in middle-aged women (mean age 50.4 years) using the Center for Epidemiological Studies Depression scale [27] and in overweight premenopausal women using the Zung's Self-Rating Depression Scale [44] reported that central obesity measured as visceral fat (but not subcutaneous fat) was significantly associated with an increased likelihood of having depression; surprisingly, waist circumference as an indicator of central obesity was not associated with depression in the study conducted by Everson-Rose et al [27]. A recent study using the PHQ-9 reported that waist circumference in the third and fourth quartiles was significantly associated with an increased likelihood of moderate-to-severe depression but not major depression [45], however, that study was conducted in U.S. adult women only, and only age- or age- and BMI-adjusted odds ratios were reported [45]. Our study using the data from both men and nonpregnant women who were overweight and obese further demonstrated that waist circumference and abdominal obesity were significantly associated with both major depressive symptoms and moderate-to-severe depressive symptoms after adjusting for multiple potential confounders. However, we did not conduct sex-stratified analyses because interactions between sex and waist circumference or abdominal obesity on outcome measures were not significant in the present study. In addition, we did not include BMI as a covariate because we only focused on people who were overweight and obese and also because of the high correlation between waist circumference and BMI [46,47]. Whether or not BMI should be included as a covariate in studies like ours or in studies dealing with metabolic syndrome [39-43] remains controversial at present.

Our stratified analyses by BMI and abdominal obesity revealed that overweight adults with abdominal obesity were more likely to have depressive symptoms (by both definitions) than overweight adults without abdominal obesity; however, there were no differences in obese adults with and without abdominal obesity. The fact that about 96% of obese adults have abdominal obesity may explain this observation. Nevertheless, our finding is consistent with previous research in obese women reported by Ma and Xiao [45]. Moreira et al.[29] reported that increasing in waist circumference was significantly associated with an increased prevalence of depressive symptoms and mood disorders in obese women; however, in that study, only simple correlation analysis was conducted and potential confounders were not taken into account. Taken together, the negligible differences in the prevalence and the odds ratios of having depressive symptoms between overweight and obese adults with abdominal obesity in the present study further suggest that waist circumference or abdominal obesity may be a preferred predictor of depression in this population.

Our study is subject to several limitations. First, the causal relationship between waist circumference or abdominal obesity and having depressive symptoms cannot be established based on the nature of our cross-sectional study. A growing body of evidence has shown that a bidirectional relationship may exist. Obesity in adolescence was associated with later depression in young adulthood [22]. The poor social relationships, low socioeconomic status, and the multiple chronic diseases associated with obesity may have predisposed obese people to impaired mental health. On the other hand, longitudinal studies have shown that baseline depression is a significant predictor of visceral fat accumulation and obesity [48-50] and is associated with increased adrenal gland volume [49]. The latter suggests a long-term increased production of stress hormones from the hypothalamic-pituitary-adrenal (HPA) axis is involved in depression, which contributes to body fat accumulation [51,52]. Second, our study was conducted only in overweight and obese participants representing a high risk population; this may have affected the generalizability of our results. Third, we conducted our analyses from combined data from both men and women mainly due to lack of interactions between sex and waist circumference or abdominal obesity on outcome measures and due to relatively small sample size. Future studies using sex-stratified data analyses are warranted to further explore sex disparities in the associations of depression with waist circumference and abdominal obesity and to study the potential effects of menopause on the associations. Fourth, we used PHQ-9 as a measure of depressive symptoms rather than a clinical diagnosis of depression. Although the PHQ-9 depression assessment has been validated in the general population including people who are overweight and obese as well as in patients with diabetes, coronary artery disease, or chronic heart failure, research on specific validation of this instrument in obese adults is currently not available. Thus, studies on clinical diagnosed depression and its association with abdominal obesity are warranted. Finally, antidepressant treatments, which are associated with weight gain [53], were not taken into account in the present study.

## Conclusions

Our study from a large nationally representative sample demonstrated that waist circumference or abdominal obesity was associated with an increased likelihood of having major or moderate-to-severe depressive symptoms among overweight and obese adults. The continuing increases in BMI and waist circumference in the United States [54,55] and the projected increases in the prevalence of overweight and obesity [1,56] suggest that mental health status should be screened, monitored, and evaluated especially in people with abdominal obesity. A routine anthropometric measure of waist circumference as a simple and practical measure of abdominal obesity may be useful for providing information on depression risk in this population.

## Competing interests

The authors declare that they have no competing interests.

## Authors' contributions

GZ obtained the data from NHANES web, conducted the data analyses, interpreted the data, and prepared the manuscript. ESF supervised the data analyses and contributed to the manuscript writing. CL, JT, SD, and LSB participated in the revisions and made critical revisions of the manuscript for important intellectual content. All authors contributed to and have approved the final manuscript.

## Pre-publication history

The pre-publication history for this paper can be accessed here:

http://www.biomedcentral.com/1471-244X/11/130/prepub
